# Why CAR T cell therapy fails in renal cell carcinoma

**DOI:** 10.3389/fimmu.2026.1828738

**Published:** 2026-06-11

**Authors:** Hua-jun Zhang, Lv-zhou Han, Xiang Zhang, Yang-lu Ge

**Affiliations:** 1 Yuyao Hospital of Traditional Chinese Medicine, Ningbo, Zhejiang, China; 2 School of Medicine, Shanghai University, Shanghai, China

**Keywords:** CAR-T cell therapy, hypoxia, metabolic reprogramming, myeloid cell suppression, renal cell carcinoma, tumor microenvironment

## Abstract

Chimeric antigen receptor (CAR) T cell therapy has transformed the treatment landscape of hematologic malignancies, yet its clinical efficacy in solid tumors remains limited. Renal cell carcinoma (RCC) presents a striking paradox: despite its established responsiveness to immune modulation and the expression of targetable tumor antigens, CAR-T therapies have failed to produce durable clinical benefit. This failure has often been attributed to antigen heterogeneity or lack of tumor specificity; however, accumulating clinical and experimental evidence suggests that antigen recognition alone does not determine therapeutic success in RCC. In this review, we discuss evidence that CAR-T failure in RCC may reflect consistent biological constraints suggesting a systemic mismatch between engineered T cells and the renal tumor ecosystem. Across clinical studies targeting multiple RCC-associated antigens, CAR-T cells demonstrate limited tumor trafficking, rapid functional decline, and poor intratumoral persistence, with little evidence of antigen-driven escape. We examine three interrelated barriers underlying this failure: immune exclusion driven by abnormal vasculature, hypoxia, and suppressive myeloid populations; profound metabolic competition and bioenergetic stress imposed by the uniquely rewired RCC microenvironment; and the insufficiency of antigen targeting in the absence of environmental support for sustained T cell function. We further discuss how these insights necessitate a shift from generic CAR-T platforms toward RCC-adapted cellular therapies. Strategies that enhance tumor homing, improve metabolic fitness, tolerate hypoxia, and actively remodel the myeloid-dominated microenvironment may be essential for achieving durable efficacy. Finally, we outline implications for clinical trial design, patient selection, and biologically rational combination strategies. Reframing CAR-T therapy as a systems-level intervention, rather than a target-restricted cytotoxic approach, may be critical for unlocking its potential in renal cell carcinoma.

## 1 Introduction: understanding why CAR T cell therapy fails in renal cell carcinoma

Renal cell carcinoma (RCC) presents a persistent paradox in cancer immunotherapy. Among solid tumors, RCC is unusually responsive to immune modulation, with durable benefit observed following immune checkpoint blockade, cytokine therapy, and rational immuno-targeted combinations (1–3). These clinical successes would, in principle, predict susceptibility to adoptive cellular therapies such as chimeric antigen receptor T (CAR-T) cells. However, despite transformative efficacy in hematologic malignancies, CAR-T therapy has failed to produce durable clinical benefit in RCC (4, 5).

This discrepancy has often been attributed to the lack of a suitable tumor-specific antigen. While antigen heterogeneity and shared expression with normal tissues are genuine challenges in RCC, this explanation is incomplete. Multiple RCC-associated antigens—including carbonic anhydrase IX (CAIX), CD70, AXL, and ROR2—are robustly expressed in defined patient subsets, and antigen-specific CAR-T engagement can be demonstrated experimentally (6–8). Nonetheless, clinical responses remain rare, transient, or absent.

The reproducible failure of CAR-T therapy across targets, constructs, and clinical contexts suggests that antigen recognition alone does not define therapeutic success in RCC. Instead, CAR-T cells appear unable to establish sustained functional activity within the renal tumor milieu (9, 10).

In this review, we propose that CAR-T failure in RCC may reflect consistent biological constraints suggesting a systemic mismatch between engineered T cells and the renal tumor ecosystem, acknowledging that clinical data remain limited and trials are early-phase and heterogeneous. Rather than a problem of target selection, RCC represents a setting in which trafficking, persistence, and effector function are collectively constrained by tumor-intrinsic and microenvironmental features. Understanding this mismatch is essential for rational redesign of CAR-T strategies tailored to RCC biology.

## 2 Clinical reality of CAR T therapy

### 2.1 Overview of clinical experience with CAR T cells

Clinical evaluation of CAR-T therapy in RCC has been limited but instructive. Early trials targeting CAIX demonstrated *in vivo* antigen recognition but were complicated by on-target, off-tumor toxicity, reflecting expression in biliary epithelium (11). Modified approaches incorporating lower-affinity receptors or regional delivery improved safety profiles but failed to induce durable tumor regression (12).

Subsequent efforts have focused on alternative targets such as CD70, which is highly expressed in RCC with restricted normal tissue distribution. These studies have generally demonstrated acceptable safety and transient CAR-T expansion; however, objective responses remain uncommon, and intratumoral persistence is limited (13). One important challenge arises from the fact that CD70 can also be expressed on activated T cells, leading to potential fratricide or self-killing of CAR-T cells, as well as antigen masking that reduces effective engagement of tumor cells. Preclinical studies have demonstrated that CD70-targeting CAR-T cells may inadvertently recognize and eliminate CD70-expressing T cells, limiting their persistence and antitumor efficacy (14). Across trials, CAR-T cells can be successfully manufactured, infused, and detected systemically, yet they do not achieve sustained dominance within tumor tissue.

### 2.2 Shared biological failure patterns across clinical trials

Despite heterogeneity in antigen selection, CAR architecture, conditioning regimens, and patient populations, clinical studies of CAR-T therapy in RCC converge on a remarkably consistent biological pattern. Infused CAR-T cells show limited accumulation within tumor tissue, undergo rapid functional decline after infusion, and exhibit poor intratumoral persistence beyond early time points (15, 16). Notably, there is little evidence of antigen-driven tumor escape, suggesting that therapeutic failure occurs before sustained selective pressure can be imposed. This failure mode distinguishes RCC from settings in which CAR-T resistance emerges only after initial efficacy and underscores the need for RCC-specific frameworks to understand and overcome the barriers to durable cellular therapy (17, 18).

## 3 Microenvironmental and metabolic barriers to CAR T cell therapy in renal cell carcinoma

**Figure 1:**
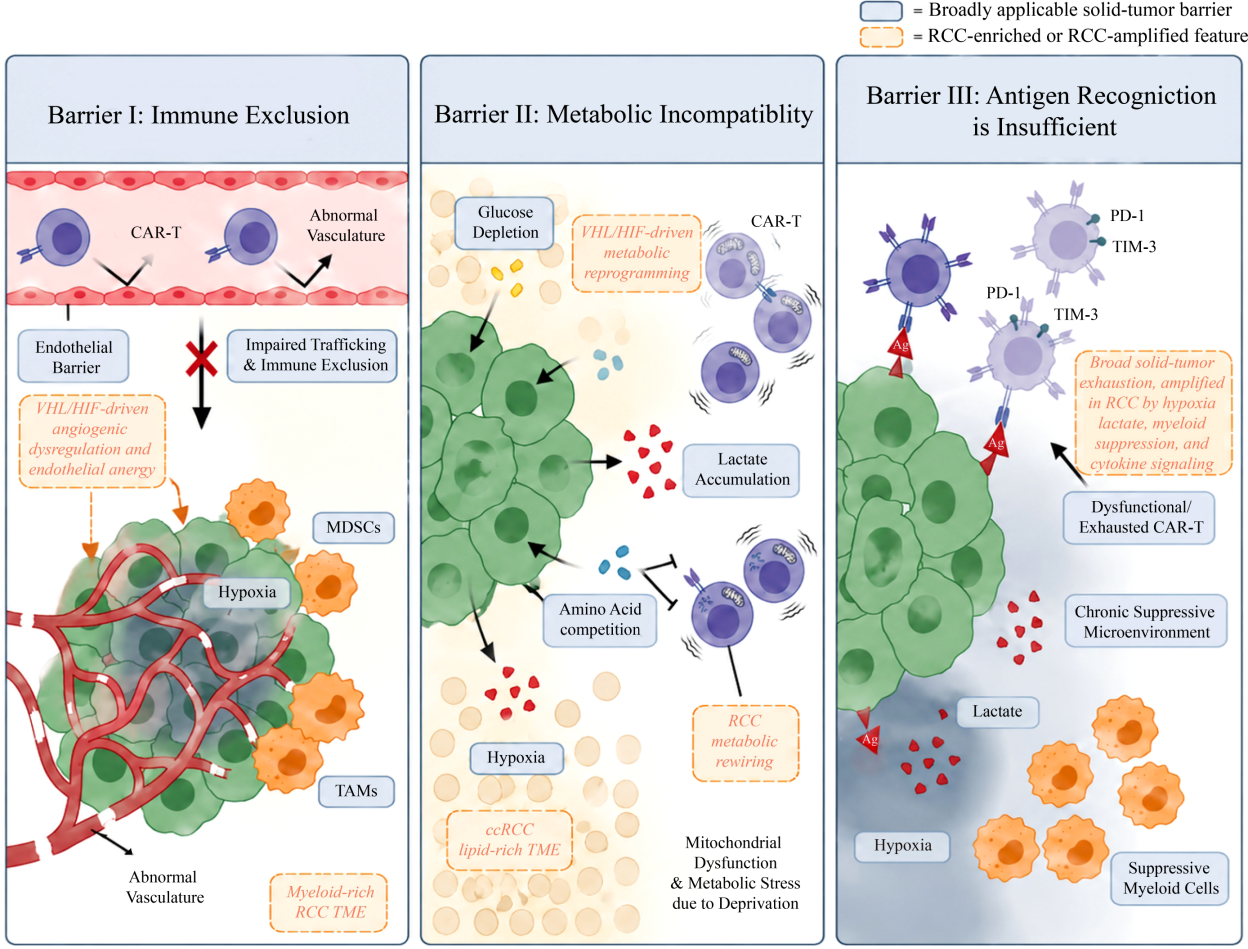
Schematic overview of the major barriers limiting the efficacy of CAR-T cell therapy in renal cell carcinoma. Blue solid boxes denote barriers broadly shared across solid tumors, whereas orange dashed boxes indicate features that are enriched or amplified in RCC. Barrier I: abnormal vasculature, endothelial barrier dysfunction/anergy, hypoxia, and suppressive myeloid populations, including MDSCs and TAMs, collectively impair CAR-T cell trafficking, infiltration, and intratumoral activity. In RCC, these effects are further reinforced by VHL/HIF-driven angiogenic dysregulation and a highly myeloid-inflamed tumor microenvironment. Barrier II: glucose deprivation, amino acid competition, lactate accumulation, hypoxia, and broader metabolic stress compromise CAR-T cell fitness and promote mitochondrial dysfunction. In clear cell RCC, these constraints are further intensified by tumor-intrinsic metabolic rewiring and a lipid-rich microenvironment. Barrier III: Although CAR-T cells can recognize and engage antigen-expressing tumor cells, antigen recognition alone is unlikely to sustain effector function or persistence in this hostile environment. Chronic exposure to hypoxia, lactate, suppressive myeloid cells, and inhibitory signaling promotes dysfunctional or exhausted states characterized by upregulation of inhibitory receptors, including PD-1 and TIM-3, thereby limiting durable antitumor efficacy.

**Figure 1** provides a conceptual framework for the biological basis of CAR-T cell failure in renal cell carcinoma by summarizing the major barriers that constrain therapeutic efficacy. These include immune exclusion, metabolic incompatibility, and the insufficiency of antigen recognition alone to maintain durable CAR-T cell effector function within the tumor microenvironment. Importantly, several of these barriers are broadly shared across solid tumors, including impaired trafficking, hypoxia, suppressive myeloid infiltration, nutrient competition, lactate accumulation, and T cell dysfunction or exhaustion. However, RCC is distinguished by the convergence and amplification of these barriers within a tumor ecosystem shaped by von Hippel-Lindau (VHL) loss, constitutive hypoxia-inducible factor (HIF) signaling, dysregulated angiogenesis, and profound metabolic reprogramming.

## 4 Barrier I: the tumor microenvironment as a zone of immune exclusion

### 4.1 Abnormal vasculature and impaired trafficking of CAR T cells

Clear cell RCC is defined by dysregulated angiogenesis driven by von Hippel–Lindau (VHL) loss and constitutive activation of hypoxia-inducible factors (HIFs). While RCC tumors are highly vascularized, this vasculature is structurally chaotic, poorly perfused, and functionally immunosuppressive (19, 20). Endothelial cells within RCC downregulate adhesion molecules necessary for lymphocyte extravasation and actively exclude effector T cells—a phenomenon termed endothelial anergy (21).

For CAR-T cells, which rely on efficient homing and tissue penetration to exert cytotoxic effects, this represents a critical early barrier. Unlike endogenous T cells primed *in situ* or expanded under chronic antigen exposure, infused CAR-T cells must rapidly traffic from circulation into tumor tissue (22–24). In RCC, the probability that sufficient numbers of CAR-T cells ever reach the tumor parenchyma may be intrinsically low.

Furthermore, RCC tumors frequently exhibit chemokine mismatches, wherein dominant chemokines such as CCL2 and CXCL8, produced by the tumor, preferentially attract myeloid cells rather than cytotoxic lymphocytes (25), a phenomenon similarly observed in other solid tumors, such as neuroblastoma, where tumor-derived CXCL1 and CXCL2 recruit myeloid-derived suppressor cells and limit CAR-T cell infiltration and functionality (26).Consequently, CAR-T cells lacking appropriate chemokine receptors, such as CXCR3, are biologically misaligned with the RCC chemotactic landscape (27).

### 4.2 Hypoxia driven immune suppression

Hypoxia is not merely a microenvironmental feature of RCC—it is a defining oncogenic driver. Persistent HIF-2α signaling shapes tumor cell metabolism, angiogenesis, and immune interactions. For T cells, hypoxia imposes profound functional consequences, including impaired proliferation, altered differentiation, and reduced cytotoxic capacity (28, 29).

CAR-T cells are typically engineered and expanded under normoxic conditions and optimized for signaling strength rather than environmental adaptability. Upon entering hypoxic RCC niches, these cells encounter transcriptional and metabolic stress for which they are poorly prepared (30, 31). Hypoxia also amplifies suppressive cytokine gradients and enhances expression of inhibitory ligands, such as increased PD-L1 on tumor (32) and immunosuppressive cytokines such as TGF-β and IL-10 (33, 34), compounding CAR-T dysfunction.

Importantly, hypoxia-induced suppression is non-antigen specific, meaning that even highly active CAR constructs are rendered ineffective once environmental thresholds are crossed (35).

### 4.3 Dominance of suppressive myeloid cells and inhibition of CAR T function

The most underappreciated barrier to CAR-T efficacy in RCC is the overwhelming dominance of suppressive myeloid populations, including tumor-associated macrophages, myeloid-derived suppressor cells, and neutrophil subsets, which function as active architects of immune exclusion rather than passive bystanders (36–38). These cells inhibit CAR-T activity through multiple redundant mechanisms—such as arginine and tryptophan depletion, production of reactive oxygen and nitrogen species, secretion of immunosuppressive cytokines, and physical sequestration of immune niches—thereby creating a multilayered suppressive network that is difficult to overcome (39–42). While clinical CAR-T trials in RCC have documented limited intratumoral persistence and rapid functional decline, direct evidence demonstrating specific interactions between CAR-T cells and suppressive myeloid populations remains limited. Nonetheless, preclinical models suggest that myeloid cells can remodel stromal architecture and vascular permeability, contributing to the functional paralysis of CAR-T cells in the myeloid-rich tumor microenvironment (43). As a result, CAR-T cells in RCC may successfully recognize their target antigen yet remain functionally paralyzed within a myeloid-dominated tumor microenvironment that precludes sustained effector function.

Suppressive myeloid populations, including tumor-associated macrophages (TAMs), myeloid-derived suppressor cells (MDSCs), and neutrophil subsets, represent a dominant barrier to CAR-T efficacy in RCC (34–36). Clinical studies in RCC patients receiving CAR-T therapy [e.g., CD70-targeted allogeneic CAR T cells (4,5)] have observed that tumors with high myeloid infiltration exhibit reduced CAR-T expansion and limited intratumoral persistence, suggesting active suppression of T cell function. Preclinical models further demonstrate that myeloid cells inhibit CAR-T activity via nutrient depletion (arginine, tryptophan), production of reactive oxygen/nitrogen species, and secretion of immunosuppressive cytokines such as TGF-β and IL-10 (35). Spatial analyses of RCC tissue reveal that CAR-T cells preferentially localize to regions with lower myeloid density, underscoring the role of myeloid cells in shaping T cell distribution and functional paralysis within the TME (35, 36). These data highlight the need for strategies that remodel the myeloid compartment to enhance CAR-T cell infiltration and persistence in RCC.

## 5 Barrier II: metabolic incompatibility between CAR-T cells and renal cell carcinoma

While immune suppression and trafficking barriers are increasingly recognized in solid tumors, RCC presents a uniquely metabolically hostile environment for adoptively transferred T cells. Clear cell RCC is among the most metabolically rewired human cancers, characterized by profound alterations in glucose, lipid, and amino acid metabolism driven by constitutive HIF signaling and mitochondrial dysfunction (44–46). These tumor-intrinsic features reshape the TME in ways that are fundamentally incompatible with CAR-T cell bioenergetic demands.

### 5.1 Nutrient competition and bioenergetic failure of CAR-T cells

Effective CAR-T function requires rapid proliferation, sustained cytokine production, and continuous cytotoxic activity—processes that impose substantial metabolic demands. Activated T cells rely heavily on glycolysis and glutaminolysis, particularly during early effector phases (47). In RCC, however, tumor cells and stromal components aggressively consume glucose and key amino acids, creating a nutrient-depleted environment that favors immune suppression (48).

RCC tumors exhibit high rates of aerobic glycolysis and lactate production, leading to local acidification of the TME. Elevated lactate levels directly impair T-cell receptor signaling, cytokine secretion, and cytolytic granule release (49, 50). Unlike endogenous tumor-infiltrating lymphocytes that may gradually adapt to these conditions, CAR-T cells are abruptly exposed to metabolic stress upon tumor entry, precipitating early functional collapse.

Moreover, amino acid depletion—particularly arginine and tryptophan mediated by myeloid-derived suppressor cells—further constrains CAR-T proliferation and survival (51–53). These metabolic checkpoints operate independently of antigen engagement, reinforcing the notion that CAR-T failure in RCC is driven by environmental insufficiency rather than signaling incompetence.

### 5.2 Lipid-rich microenvironment and mitochondrial stress

A defining feature of clear cell RCC is aberrant lipid accumulation, reflected in both tumor histology and transcriptional programs. RCC cells exhibit enhanced lipid uptake, storage, and utilization, resulting in a TME enriched in free fatty acids and oxidized lipids (54, 55). While some memory T-cell subsets can utilize fatty acid oxidation, excessive lipid exposure induces lipotoxicity, mitochondrial stress, and oxidative damage in effector T cells (56).

CAR-T cells, particularly those engineered with strong costimulatory signaling domains, are prone to mitochondrial exhaustion under conditions of chronic metabolic stress. Accumulation of dysfunctional mitochondria compromises ATP generation, increases reactive oxygen species, and accelerates T-cell exhaustion. These effects are exacerbated in hypoxic environments, where mitochondrial respiration is already constrained (57–59).

Notably, current CAR-T manufacturing protocols prioritize rapid expansion and effector differentiation, often at the expense of mitochondrial fitness and metabolic flexibility (60). As a result, infused CAR-T cells may be intrinsically ill-equipped to tolerate the lipid-rich, hypoxic conditions characteristic of RCC.

## 6 Necessary but not sufficient: limitations of antigen targeting

RCC-associated antigens, including CAIX and CD70, are expressed at levels sufficient for CAR engagement, and CAR-T cells targeting these antigens demonstrate antigen-specific cytotoxicity *in vitro* and, in some cases, *in vivo (*14, 61). However, these interactions rarely result in durable clinical responses, indicating that antigen recognition alone is insufficient in a tumor environment that does not support sustained CAR-T cell function. Preclinical data suggest that even low-to-moderate antigen density can trigger CAR signaling (62), yet clinical efficacy remains constrained by microenvironmental barriers such as immune exclusion, hypoxia, metabolic stress, and suppressive myeloid populations, which collectively limit infiltration, persistence, and cytotoxic function (9, 63).

Evidence for antigen loss as a resistance mechanism in RCC is limited, largely because CAR-T cells fail to persist long enough to impose sustained selective pressure (64–66). Spatial analyses of RCC tumors reveal that antigen-positive cells often coexist with immune-excluded regions and suppressive stromal compartments (67–69). CAR-T cells encountering antigen at the tumor periphery may be rapidly rendered dysfunctional before penetrating deeper tumor niches. In this context, antigen heterogeneity appears as a downstream consequence of CAR-T failure rather than its primary cause.

### 6.1 Implications for CAR design and targeting strategies

Recognizing that antigen targeting is necessary but insufficient has important implications for CAR-T development in RCC. Multispecific or logic-gated CARs may mitigate antigen escape but will not overcome barriers related to trafficking, metabolism, or myeloid suppression. Similarly, increasing CAR signaling strength may exacerbate exhaustion without improving persistence (70).

These considerations argue for a shift in emphasis: from identifying the “perfect” antigen to engineering CAR-T cells capable of surviving and functioning within the RCC ecosystem. Antigen selection should be integrated into a broader design framework that prioritizes environmental adaptability and sustained effector competence.

## 7 Re-engineering CAR-T cells for renal cell carcinoma: from generic platforms to tumor-adapted therapies

**Figure 2:**
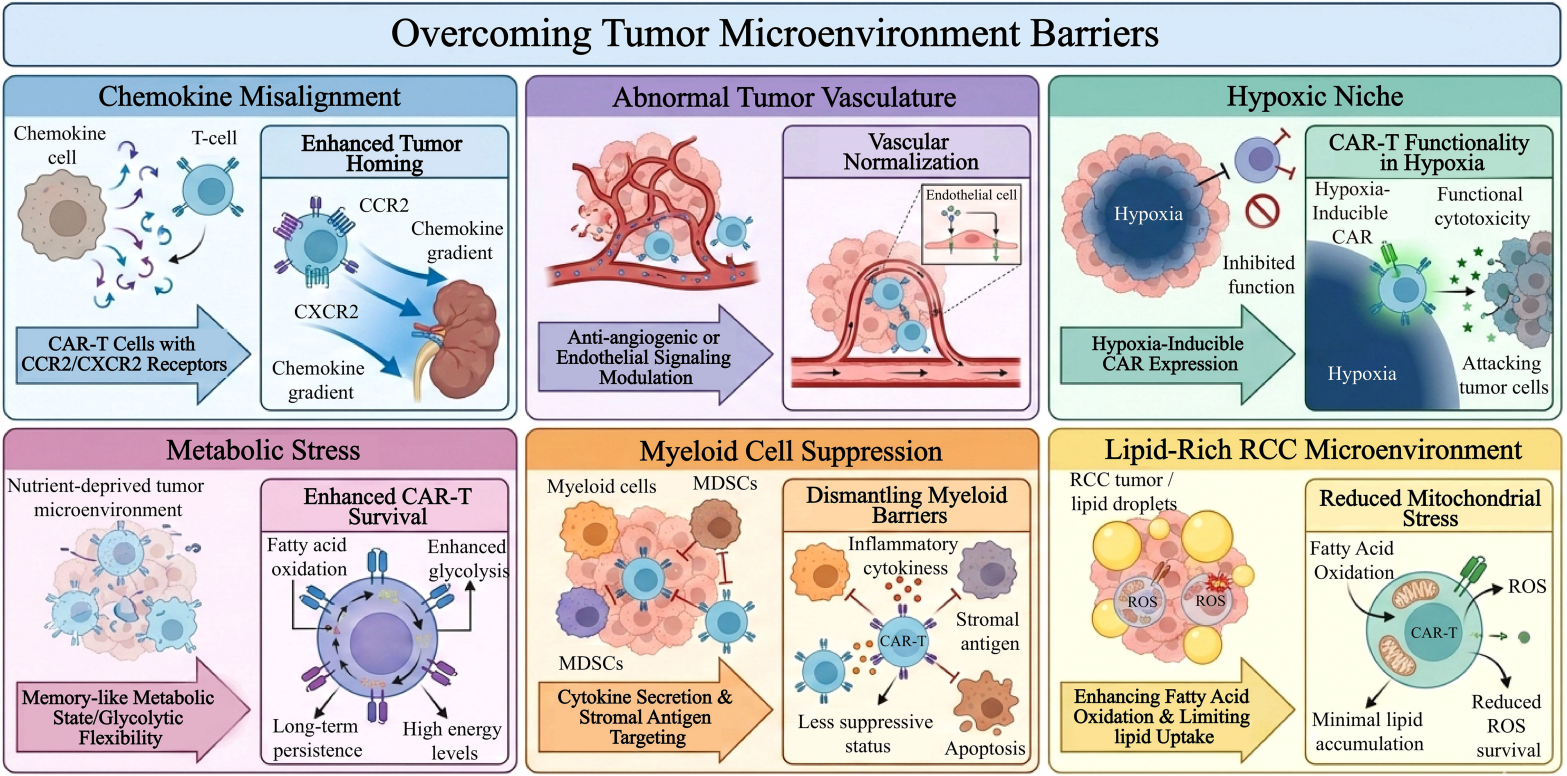
Enhanced CAR-T therapy strategies in RCC. To improve CAR-T cell therapy in RCC by addressing key tumor microenvironment barriers, chemokine misalignment can be overcome by engineering CAR-T cells with CCR2/CXCR2 receptors to enhance tumor homing. Abnormal tumor vasculature can be targeted with vascular normalization strategies to improve T-cell infiltration. Hypoxic conditions in RCC tumors can be bypassed by using hypoxia-inducible CAR expression to maintain CAR-T cell function. Metabolic stress in the RCC microenvironment can be mitigated by reprogramming CAR-T cells to a memory-like metabolic state, improving their persistence and energy balance. Finally, myeloid cell suppression can be overcome by targeting cytokine secretion and stromal antigens in myeloid cells, while lipid-rich conditions in RCC can be managed by enhancing fatty acid oxidation and limiting lipid uptake to reduce mitochondrial stress and prolong CAR-T cell survival.

If CAR-T failure in RCC is driven by microenvironmental hostility and metabolic incompatibility, then incremental modifications to existing CAR constructs are unlikely to suffice. Instead, RCC demands a context-aware redesign of CAR-T therapy, in which cellular engineering is guided by the biological constraints of the renal tumor ecosystem. The **Figure 2** depicts strategies for re-engineering CAR-T cells to address challenges in the RCC tumor microenvironment, focusing on tumor homing, metabolic adaptation, and overcoming myeloid suppression to enhance CAR-T efficacy. Hypoxic conditions, a defining feature of RCC, can be specifically addressed using hypoxia-inducible CAR systems, such as HIF-responsive promoters or the HITA system, which enable CAR expression under oxygen levels below 2% and preserve cytotoxic activity and proliferation in hypoxic niches (71, 72). These modifications are crucial for navigating RCC’s hostile environment.

### 7.1 Strategies to enhance trafficking and tumor entry

Given the central role of defective trafficking in RCC, improving CAR-T access to tumor tissue represents a foundational requirement for efficacy.

One promising strategy involves chemokine receptor engineering. RCC tumors commonly secrete chemokines such as CCL2, CXCL8, and CXCL12 that preferentially recruit myeloid cells rather than cytotoxic lymphocytes (73). Engineering CAR-T cells to express corresponding chemokine receptors (e.g., CCR2, CXCR2) may partially overcome this mismatch and enhance tumor homing (74–76). Similar trafficking limitations and chemokine mismatches have also been described in other solid tumors, including diffuse intrinsic pontine glioma (DIPG) and neuroblastoma, and have been addressed by ectopic expression of chemokine receptors on CAR-T cells (26, 27).

Complementary approaches include transient vascular normalization, either through anti-angiogenic agents or targeted modulation of endothelial signaling pathways, to improve immune cell extravasation (77, 78). However, such strategies must be carefully timed, as prolonged vascular disruption may further exacerbate hypoxia and immune exclusion.

Importantly, enhanced trafficking alone is insufficient; CAR-T cells must also survive and function once they enter the tumor. Thus, trafficking strategies should be viewed as enabling steps rather than standalone solutions.

Similar trafficking limitations and chemokine mismatches have also been described in other solid tumors, including diffuse intrinsic pontine glioma (DIPG) and neuroblastoma, and have been addressed by ectopic expression of chemokine receptors on CAR-T cells(25, Giudice et al., 2025). Complementary approaches include transient vascular normalization, either through anti-angiogenic agents or targeted modulation of endothelial signaling pathways, to improve immune cell extravasation (71, 72). However, such strategies must be carefully timed, as prolonged vascular disruption may further exacerbate hypoxia and immune exclusion. Importantly, enhanced trafficking alone is insufficient; CAR-T cells must also survive and function once they enter the tumor. Thus, trafficking strategies should be viewed as enabling steps rather than standalone solutions.

### 7.2 Metabolically adapted and stress resistant CAR T cells

The metabolic fragility of CAR-T cells in RCC suggests that bioenergetic fitness should be elevated to a core design principle. Several engineering strategies are particularly relevant in this context.

First, CAR constructs can be optimized to favor memory-like metabolic states, characterized by enhanced mitochondrial mass, oxidative phosphorylation capacity, and resistance to exhaustion (59, 79). In particular, CAR-T cells incorporating 4-1BB co-stimulatory domains demonstrate greater mitochondrial biogenesis, enhanced fatty acid oxidation, and improved persistence compared with CD28-based CARs, which preferentially drive glycolytic metabolism and terminal effector differentiation (80). Preclinical studies have further shown that IL-15-engineered CAR-T cells maintain stem cell memory phenotypes and improve mitochondrial fitness under chronic metabolic stress (81). Modulating costimulatory domains or manufacturing conditions to preserve mitochondrial integrity may improve persistence in metabolically hostile environments (82, 83).

Second, CAR-T cells may be engineered to tolerate or exploit hypoxia. Hypoxia-inducible CAR expression systems, oxygen-sensitive signaling switches, or enhanced glycolytic flexibility could allow CAR-T cells to maintain functionality within RCC niches where oxygen tension is persistently low (71, 72, 84). For example, hypoxia-inducible CAR systems such as HiCAR and the HITA platform selectively activate CAR expression under hypoxic conditions while preserving cytotoxicity and reducing off-tumor toxicity in preclinical solid tumor models (85). Additional experimental studies have shown that metabolically enhanced CAR-T cells with improved glycolytic flexibility retain proliferation and effector activity under severe hypoxia (86).

Third, strategies to mitigate lipid-induced toxicity—such as enhancing fatty acid oxidation control or limiting lipid uptake—may reduce mitochondrial stress and prolong CAR-T survival in lipid-rich RCC microenvironments (87). Preclinical studies have demonstrated that inhibition of CD36-mediated lipid uptake can reduce ferroptosis, preserve mitochondrial integrity, and restore intratumoral CD8+ T-cell function in lipid-rich tumors (88). These findings may be particularly relevant in clear cell RCC, which is characterized by profound lipid accumulation and altered fatty acid metabolism.

### 7.3 Targeting the myeloid axis to remodel the tumor microenvironment

Given the dominant suppressive role of myeloid cells in RCC, an emerging strategy is to repurpose CAR-T cells as active modulators of the tumor microenvironment, rather than as tumor cell–restricted killers.

Approaches under investigation include CAR-T cells engineered to secrete cytokines or biologics that reprogram myeloid populations, disrupt suppressive signaling networks, or promote immune-permissive stromal remodeling (89). For example, IL-7/CCL19-secreting CAR-T cells have been shown in preclinical solid tumor models to enhance dendritic cell recruitment, improve endogenous T-cell infiltration, and prolong CAR-T persistence within tumors (90). Similarly, armored macrophage-targeted CAR-T cells were recently shown to reprogram suppressive tumor-associated macrophages into pro-inflammatory phenotypes and substantially remodel the tumor microenvironment in metastatic cancer models (43).

Alternatively, CAR-T cells may be directed against stromal or myeloid-associated antigens, indirectly dismantling the barriers that prevent effective antitumor immunity (91). In breast cancer models, selective targeting of myeloid-derived suppressor cells enhanced CAR-T expansion and restored antitumor activity (39). Similar myeloid-mediated resistance mechanisms have also been described in pancreatic cancer, glioblastoma, and neuroblastoma, where reversal of suppressive myeloid signaling improved CAR-T infiltration and delayed exhaustion (92–94). These observations suggest that targeting the myeloid axis may represent a broadly applicable strategy across solid tumors rather than an RCC-specific phenomenon.

Such strategies carry inherent risks, including off-tumor effects and amplified toxicity, but they directly address a primary driver of CAR-T dysfunction in RCC. Importantly, this paradigm reframes CAR-T therapy as a systems-level intervention, capable of reshaping tumor ecology rather than simply eliminating antigen-positive cells.

## 8 Implications for clinical trial design in RCC

The biological insights outlined above have direct consequences for how CAR-T therapies are evaluated in RCC. Many prior trials may have been biologically underpowered—not due to CAR design flaws, but because trial design failed to account for RCC-specific constraints.

### 8.1 Patient selection and disease context

Late-stage, heavily pretreated RCC patients represent a particularly challenging population for CAR-T therapy. Prior exposure to ICIs, TKIs, and cytotoxic agents can further distort immune landscapes, exacerbate myeloid dominance, and impair T-cell fitness. While the eventual goal may be to introduce CAR-T cells earlier in therapy, significant ethical considerations must be acknowledged, as early-phase experimental therapies may expose patients to unproven risks when effective standard-of-care options exist. Within this context, carefully defining “biologically selected” patients offers a potential strategy to identify those most likely to benefit from CAR-T intervention while minimizing risk. For example, patients with immune-inflamed tumors, lower myeloid burden, favorable metabolic profiles, or biomarkers indicative of vascular normalization may represent a biologically permissive environment for CAR-T cell activity. Selecting patients based on such mechanistic and microenvironmental features may improve therapeutic efficacy and safety while adhering to ethical standards.

Biomarkers reflecting vascular normalization, metabolic state, or myeloid infiltration may be more informative than antigen expression alone when selecting patients likely to benefit from CAR-T therapy (95).

### 8.2 Biologically rational combination strategies

Combination approaches are often proposed to rescue CAR-T efficacy in solid tumors, yet many combinations in RCC are empirically chosen rather than biologically rational. For example, concurrent administration of ICIs may exacerbate CAR-T exhaustion, while untimed anti-angiogenic therapy may worsen hypoxia and immune exclusion.

To address these challenges, preconditioning strategies such as low-dose cyclophosphamide, fludarabine, or lymphodepletion regimens can transiently reduce suppressive myeloid populations and create a more permissive environment for CAR-T expansion (96). Additionally, combination partners that modulate the tumor microenvironment have shown promise in preclinical and early clinical studies, including anti-VEGFR or anti-Ang2 agents for vascular normalization, cytokine-secreting CAR-T cells (e.g., IL-7/CCL19) to enhance immune infiltration, and targeted myeloid modulators such as CSF1R inhibitors or TRAIL-R2 CAR-T to reduce MDSC/TAM-mediated suppression (77, 89, 97).

Instead of concurrent administration, temporal sequencing—preconditioning followed by CAR-T infusion and then adjunctive microenvironmental modulation—may maximize CAR-T expansion and function while minimizing toxicity. Critically, combination partners should be selected based on their ability to alleviate specific RCC-associated barriers (vascular, metabolic, or myeloid-driven) rather than their independent cytotoxic activity.

### 8.3 Redefining clinical and biological endpoints

In early-phase clinical trials, patient safety remains the paramount endpoint. While biological metrics such as intratumoral CAR-T persistence, metabolic fitness, and microenvironmental remodeling are valuable for understanding therapeutic potential and guiding future trial design, they should be treated as correlative or exploratory endpoints rather than primary outcomes. These data are critical for justifying new trials and refining CAR-T strategies; however, biological activity alone, without evidence of clinical benefit, is not ethically sufficient to maintain an ongoing trial, as it could expose patients to potentially futile therapy. Therefore, early-phase RCC CAR-T studies should prioritize safety and any observable clinical responses, using biological endpoints to support mechanistic insights and inform iterative optimization.

## 9 Conclusions

The limited clinical success of CAR T cell therapy in renal cell carcinoma appears to reflect consistent biological constraints suggesting a systemic mismatch between current CAR-T platforms and the renal tumor microenvironment, rather than inadequate antigen targeting, although clinical evidence remains early-phase and heterogeneous. Across clinical studies, CAR-T cells show limited tumor infiltration, rapid functional decline, and poor persistence regardless of antigen choice, indicating dominant microenvironmental constraints. Vascular immune exclusion, hypoxia, myeloid-driven suppression, and metabolic competition collectively prevent sustained CAR-T activity and limit selective pressure, explaining the relative absence of antigen escape in RCC.

These observations argue for a shift from target-centric CAR-T design toward tumor-adapted cellular engineering strategies that prioritize trafficking, metabolic resilience, and microenvironmental modulation. Accordingly, clinical evaluation frameworks should emphasize biological permissiveness and mechanistic endpoints over antigen expression alone. RCC thus highlights the need for context-aware CAR-T therapies tailored to solid tumor ecosystems.
